# Supplementary material for: Investigation of the Antitumor Effects of Tamoxifen and Its Ferrocene-Linked Derivatives on Pancreatic and Breast Cancer Cell Lines
Source: Pharmaceuticals (Basel). 2022 Mar 5;15(3):314. doi: 10.3390/ph15030314 (PMC8950591; doi:10.3390/ph15030314)
Supplement: Supplementary file 1 [file pharmaceuticals-15-00314-s001.zip › Figure S5.pdf]

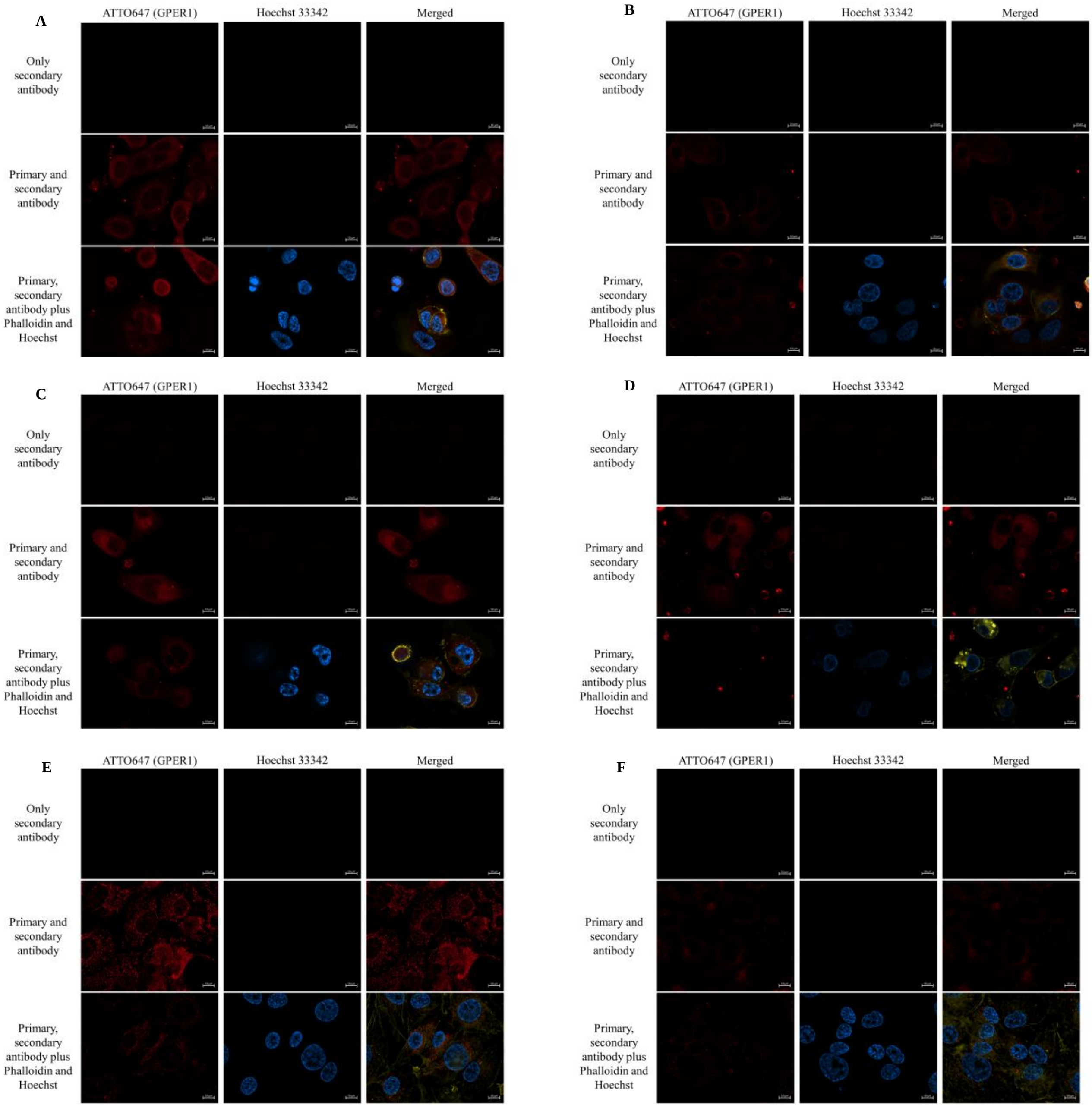

Figure S5: Comparison of the GPER1 expression of MCF7 (A: after Triton lysis, B: without lysis), MDA-MB-231 (C: after Triton lysis, D: without lysis) and PANC1 (E: after Triton lysis, F: without lysis) between Triton lysed and intact samples. Images were captured by Celldiscoverer 7. Magnification is at 100x, the scale bar is 10 µm.
