# Supplementary material for: Investigation of the Antitumor Effects of Tamoxifen and Its Ferrocene-Linked Derivatives on Pancreatic and Breast Cancer Cell Lines
Source: Pharmaceuticals (Basel). 2022 Mar 5;15(3):314. doi: 10.3390/ph15030314 (PMC8950591; doi:10.3390/ph15030314)
Supplement: Supplementary file 1 [file pharmaceuticals-15-00314-s001.zip › Figure S2.pdf]

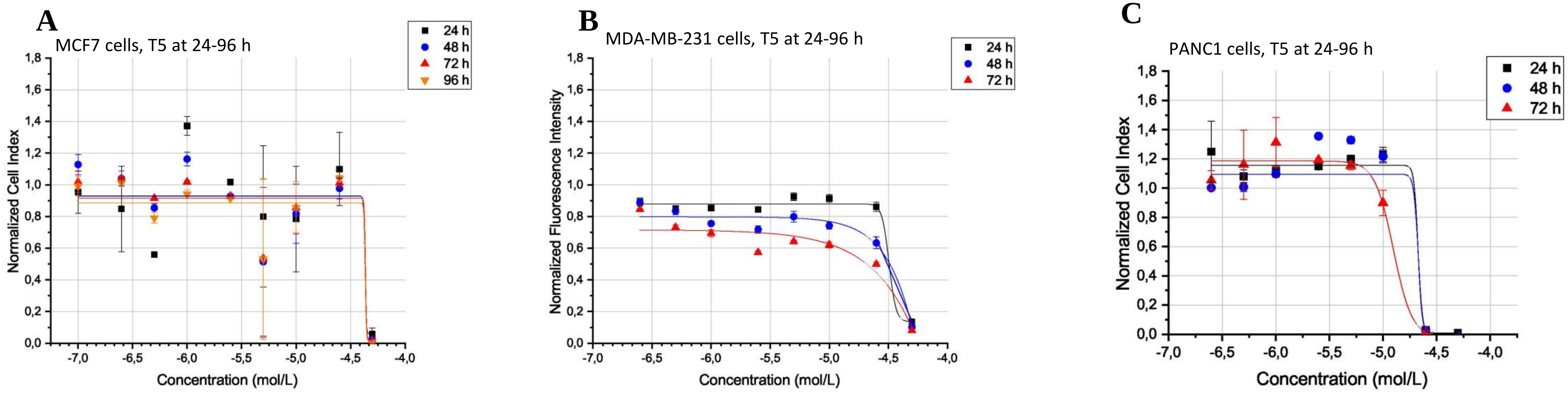

Figure S2: IC_{50} values of T5 on MCF7 (A), MDA-MB-231 (B) and PANC1 (C) cells. Each cell line was treated with concentrations between 250 nM and 25 μM (X-axis, $\log_{10}$ scale). With each concentration, three parallel samples of the same cells were measured (n=3). Data are given as mean values ± standard deviation (SD).
