# Supplementary material for: Investigation of the Antitumor Effects of Tamoxifen and Its Ferrocene-Linked Derivatives on Pancreatic and Breast Cancer Cell Lines
Source: Pharmaceuticals (Basel). 2022 Mar 5;15(3):314. doi: 10.3390/ph15030314 (PMC8950591; doi:10.3390/ph15030314)
Supplement: Supplementary file 1 [file pharmaceuticals-15-00314-s001.zip › Figure S4.pdf]

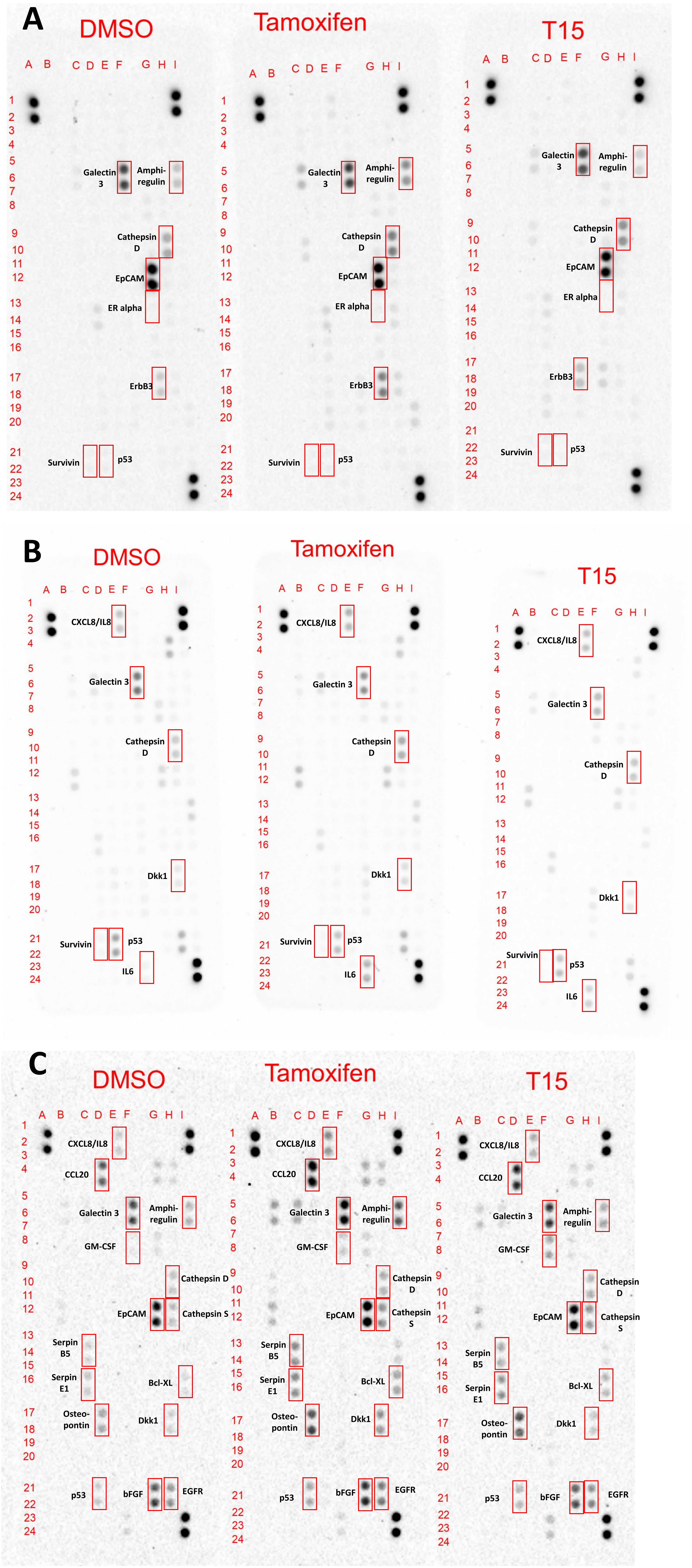

Figure S4: Pictures of the Proteome Profiler membranes of tamoxifen and T15 treated samples (A: MCF7, B: MDA-MB-231 and C: PANC1). The photos were taken with the BioRad ChemiDoc XRS+ system after 10 minutes of exposure. Proteins with 20% of the intensity measured at reference spots (at the top left and right and bottom-right corners of the membrane) were annotated.
