# Supplementary material for: Investigation of the Antitumor Effects of Tamoxifen and Its Ferrocene-Linked Derivatives on Pancreatic and Breast Cancer Cell Lines
Source: Pharmaceuticals (Basel). 2022 Mar 5;15(3):314. doi: 10.3390/ph15030314 (PMC8950591; doi:10.3390/ph15030314)
Supplement: Supplementary file 1 [file pharmaceuticals-15-00314-s001.zip › Figure S3.pdf]

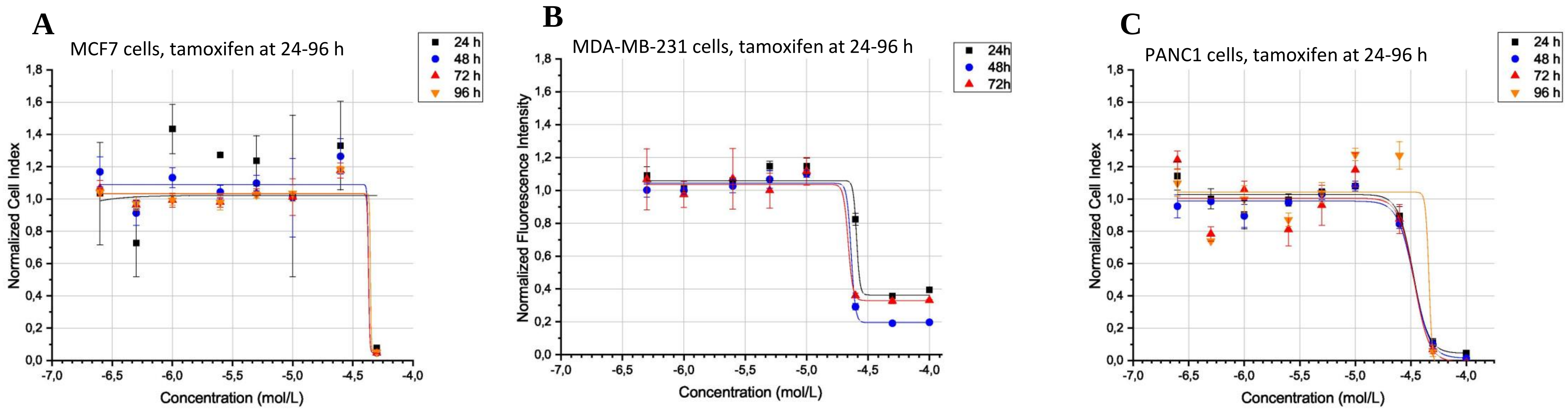

Figure S3: IC_{50} values of T15 on MCF7 (A), MDA-MB-231 (B) and PANC1 (C) cells. Each cell line was treated with concentrations between 250 nM and 25 μM (X-axis, log_{10} scale). With each concentration, three parallel samples of the same cells were measured (n=3). Data are given as mean values ± standard deviation (SD).

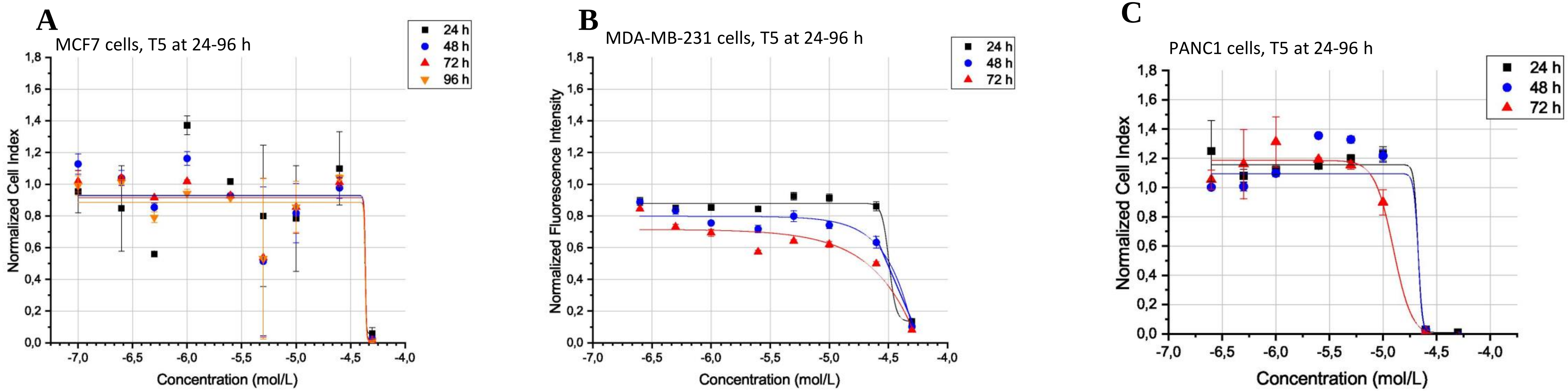
A
MCF7 cells, T5 at 24-96 h
24 h
48 h
72 h
96 h
Normalized Cell Index
Concentration (mol/L)
B
MDA-MB-231 cells, T5 at 24-96 h
24 h
48 h
72 h
Normalized Fluorescence Intensity
Concentration (mol/L)
C
PANC1 cells, T5 at 24-96 h
24 h
48 h
72 h
Normalized Cell Index
Concentration (mol/L)

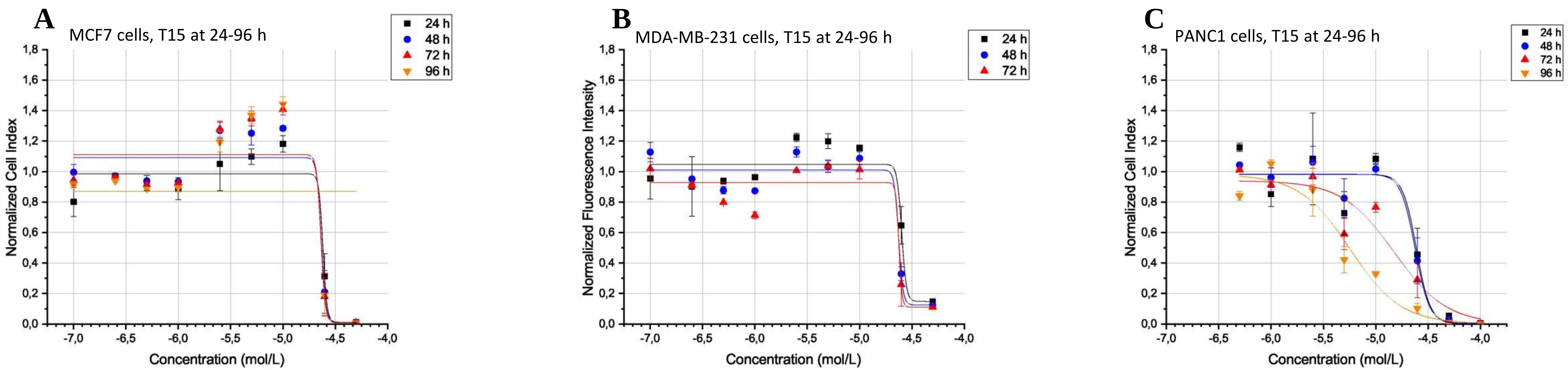
A
MCF7 cells, T15 at 24-96 h
24 h
48 h
72 h
96 h
Normalized Cell Index
Concentration (mol/L)
B
MDA-MB-231 cells, T15 at 24-96 h
24 h
48 h
72 h
Normalized Fluorescence Intensity
Concentration (mol/L)
C
PANC1 cells, T15 at 24-96 h
24 h
48 h
72 h
96 h
Normalized Cell Index
Concentration (mol/L)

A

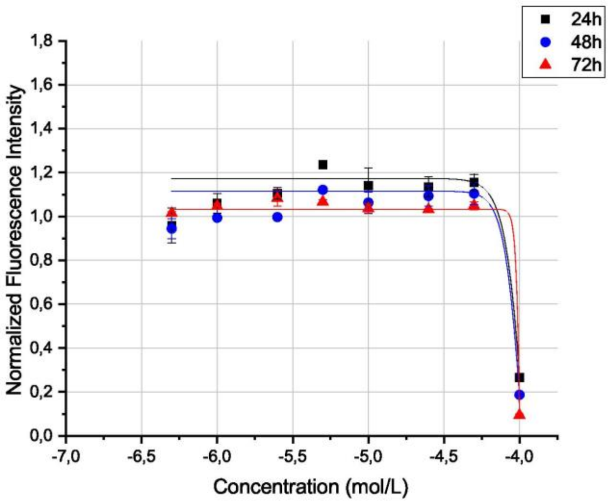
24h
48h
72h
Normalized Fluorescence Intensity
Concentration (mol/L)

B

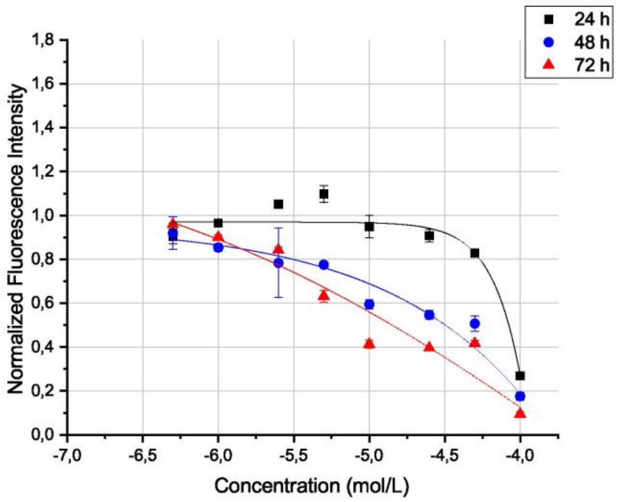
24 h
48 h
72 h
Normalized Fluorescence Intensity
Concentration (mol/L)

C

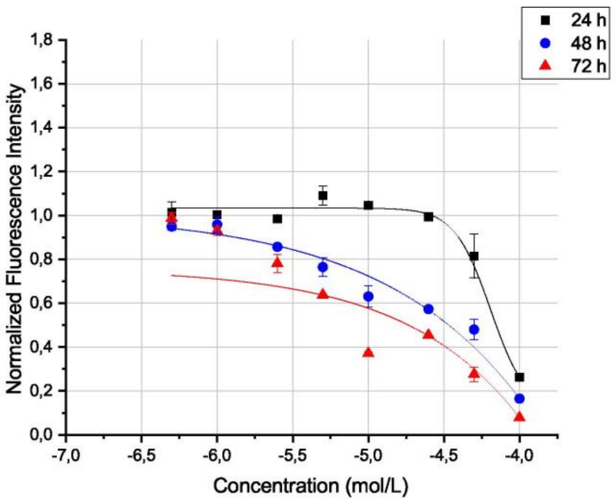
24 h
48 h
72 h
Normalized Fluorescence Intensity
Concentration (mol/L)

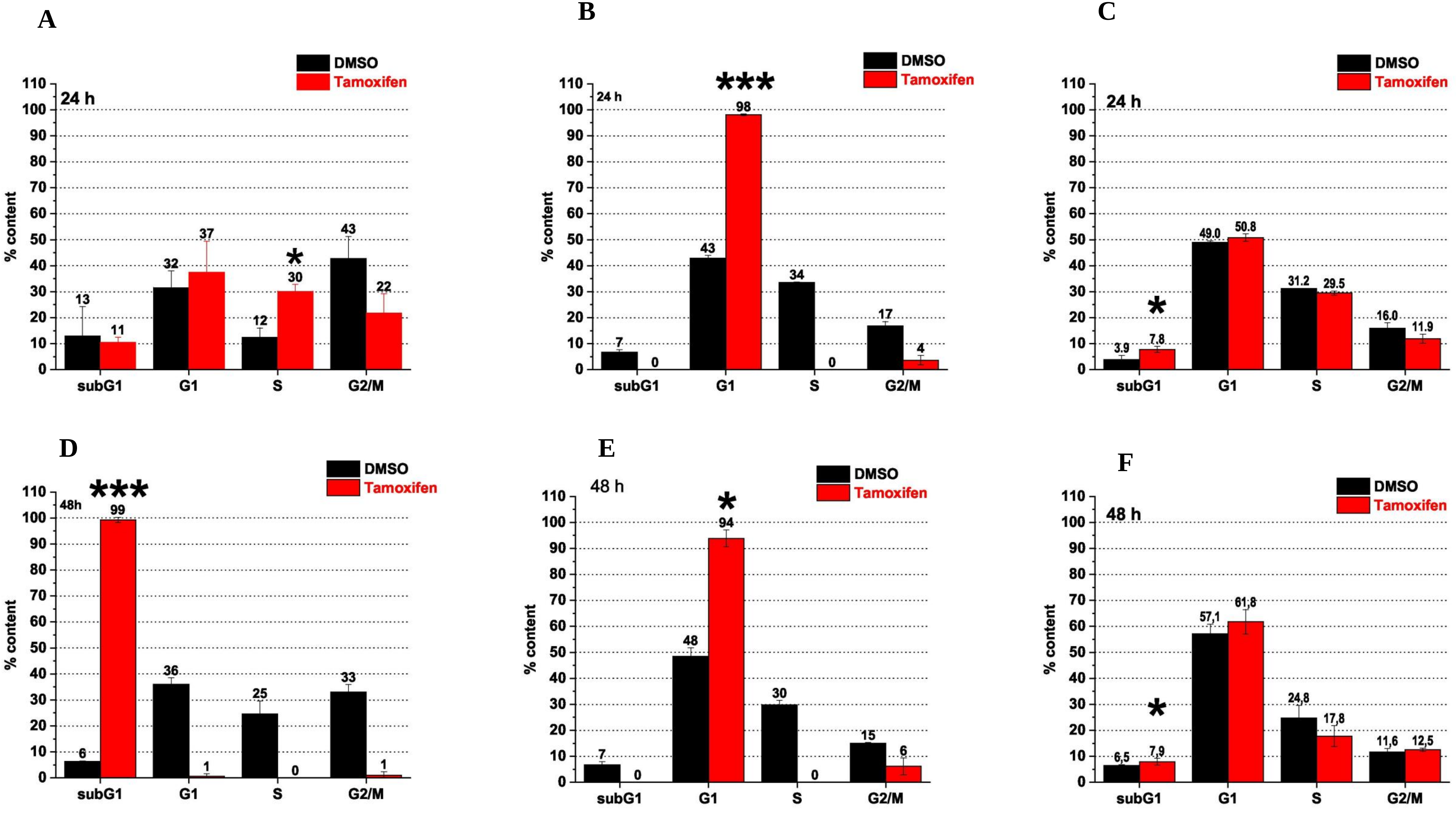
A
DMSO
Tamoxifen
24 h
% content
subG1
G1
S
G2/M
13
11
32
37
12
30
43
22
*
B
DMSO
Tamoxifen
24 h
***
% content
7
0
43
98
34
0
17
4
subG1
G1
S
G2/M
C
DMSO
Tamoxifen
24 h
% content
3.9
7.8
49.0
50.8
31.2
29.5
16.0
11.9
*
subG1
G1
S
G2/M
D
DMSO
Tamoxifen
48h
***
% content
6
99
36
1
25
0
33
1
subG1
G1
S
G2/M
E
DMSO
Tamoxifen
48 h
*
% content
7
0
48
94
30
0
15
6
subG1
G1
S
G2/M
F
DMSO
Tamoxifen
48 h
% content
6,5
7,9
57,1
61,8
24,8
17,8
11,6
12,5
*
subG1
G1
S
G2/M

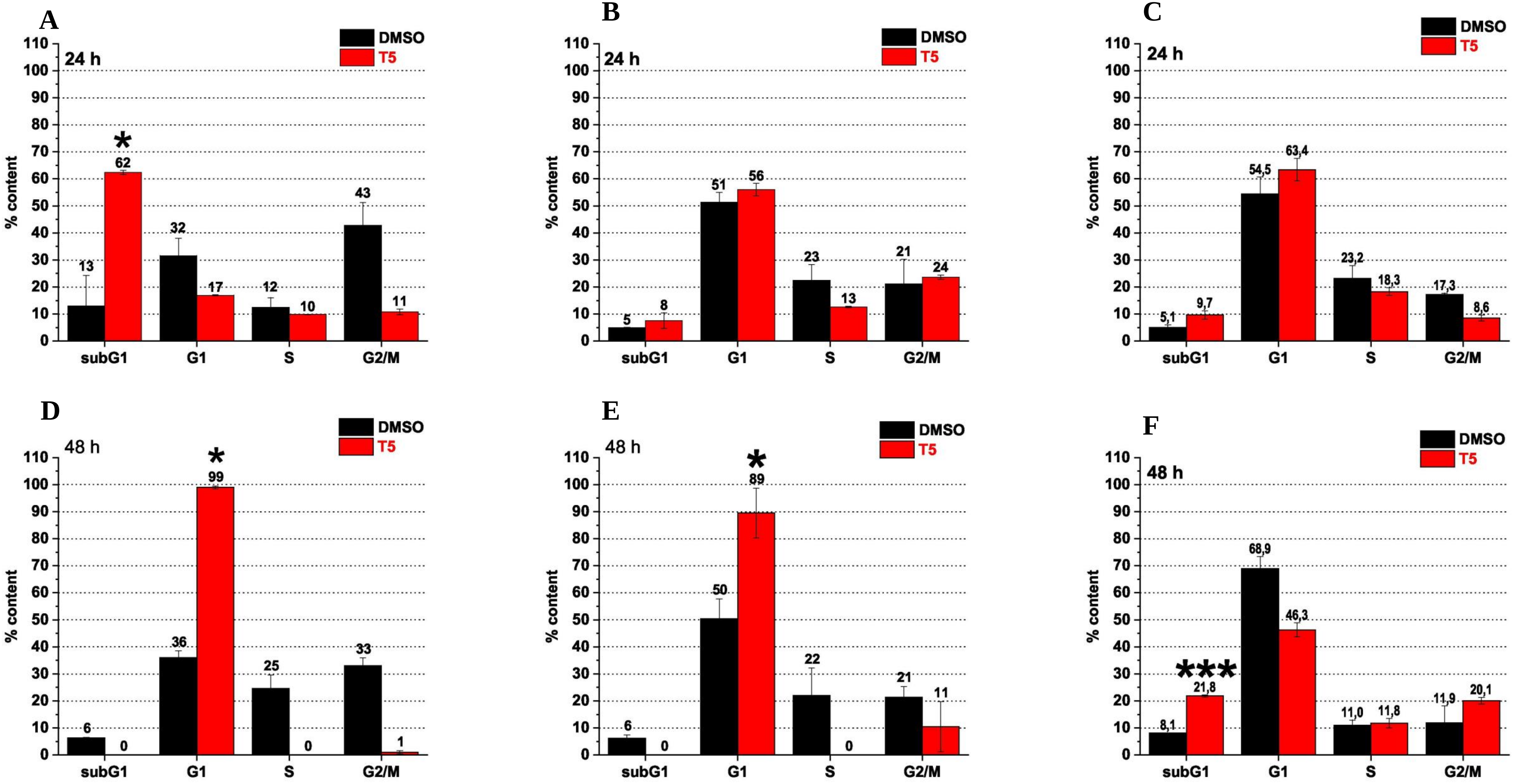
A
DMSO
T5
24 h
% content
*
62
13
32
17
12
10
43
11
subG1
G1
S
G2/M
B
24 h
5
8
51
56
23
13
21
24
C
24 h
5,1
9,7
54,5
63,4
23,2
18,3
17,3
8,6
D
48 h
*
99
6
0
36
25
0
33
1
E
48 h
*
89
6
0
50
22
0
21
11
F
48 h
***
8,1
21,8
68,9
46,3
11,0
11,8
11,9
20,1

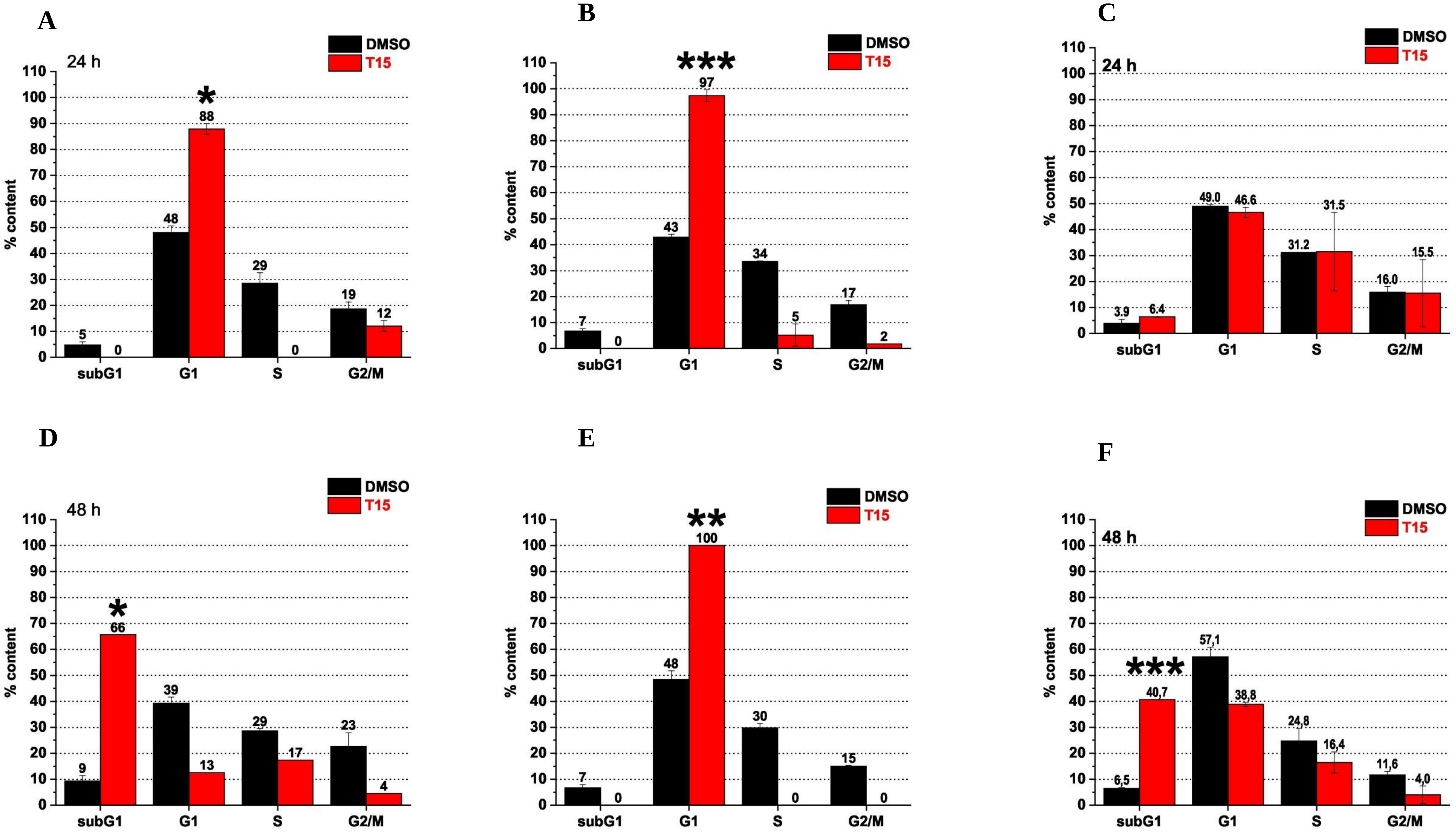
A
24 h
DMSO
T15
% content
subG1
G1
S
G2/M
5
0
48
88
*
29
0
19
12
B
DMSO
T15
% content
***
7
0
43
97
34
5
17
2
subG1
G1
S
G2/M
C
24 h
DMSO
T15
% content
3.9
6.4
49.0
46.6
31.2
31.5
16.0
15.5
subG1
G1
S
G2/M
D
48 h
DMSO
T15
% content
9
66
*
39
13
29
17
23
4
subG1
G1
S
G2/M
E
DMSO
T15
% content
7
0
48
100
**
30
0
15
0
subG1
G1
S
G2/M
F
48 h
DMSO
T15
% content
6,5
40,7
***
57,1
38,8
24,8
16,4
11,6
4,0
subG1
G1
S
G2/M

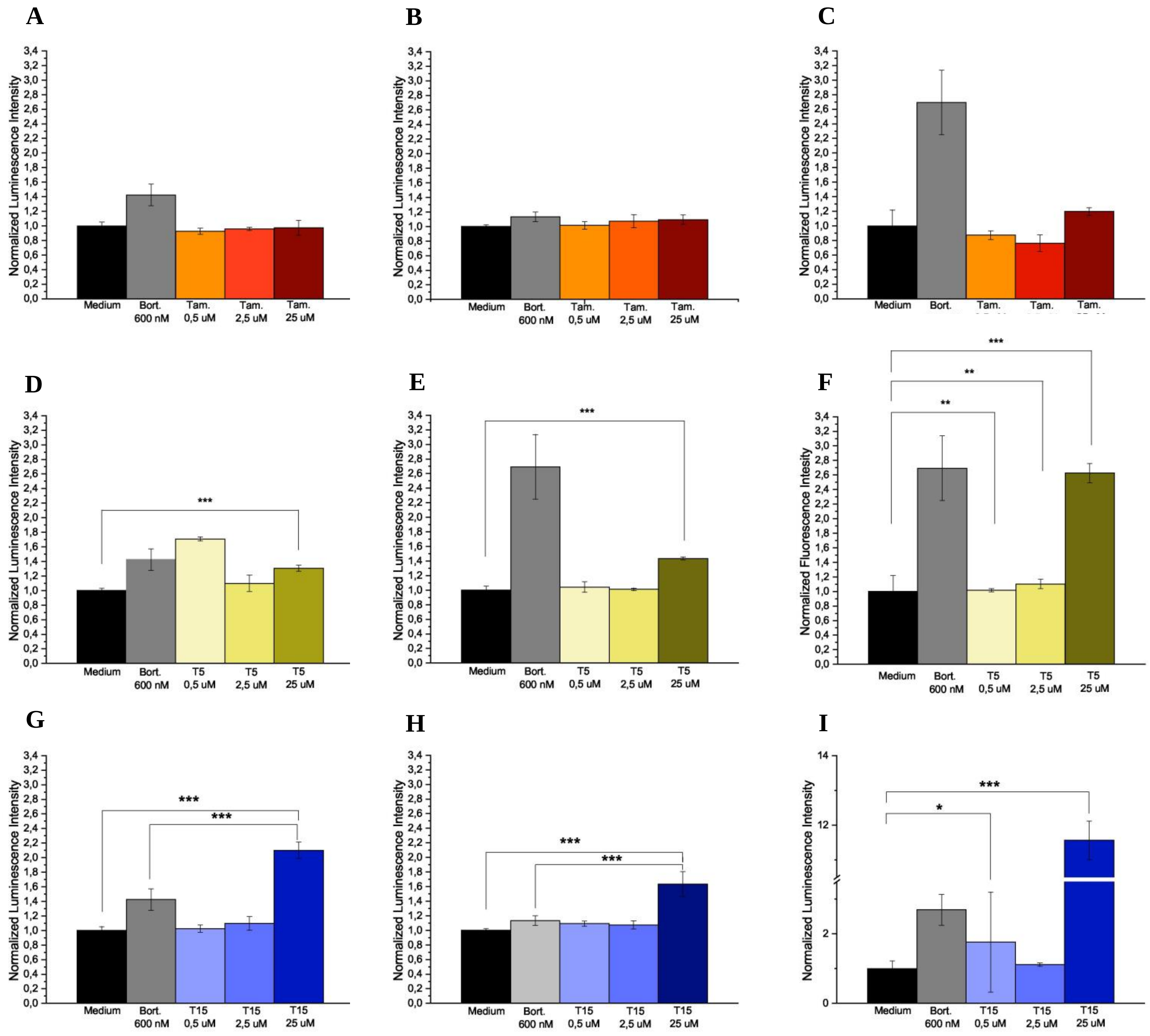
A
Normalized Luminescence Intensity
Medium
Bort. 600 nM
Tam. 0,5 uM
Tam. 2,5 uM
Tam. 25 uM
B
Normalized Luminescence Intensity
Medium
Bort. 600 nM
Tam. 0,5 uM
Tam. 2,5 uM
Tam. 25 uM
C
Normalized Luminescence Intensity
Medium
Bort.
Tam.
Tam.
Tam.
D
Normalized Luminescence Intensity
***
Medium
Bort. 600 nM
T5 0,5 uM
T5 2,5 uM
T5 25 uM
E
Normalized Luminescence Intensity
***
Medium
Bort. 600 nM
T5 0,5 uM
T5 2,5 uM
T5 25 uM
F
Normalized Fluorescence Intesity
***
**
**
Medium
Bort. 600 nM
T5 0,5 uM
T5 2,5 uM
T5 25 uM
G
Normalized Luminescence Intensity
***
***
Medium
Bort. 600 nM
T15 0,5 uM
T15 2,5 uM
T15 25 uM
H
Normalized Luminescence Intensity
***
***
Medium
Bort. 600 nM
T15 0,5 uM
T15 2,5 uM
T15 25 uM
I
Normalized Luminescence Intensity
***
*
Medium
Bort. 600 nM
T15 0,5 uM
T15 2,5 uM
T15 25 uM

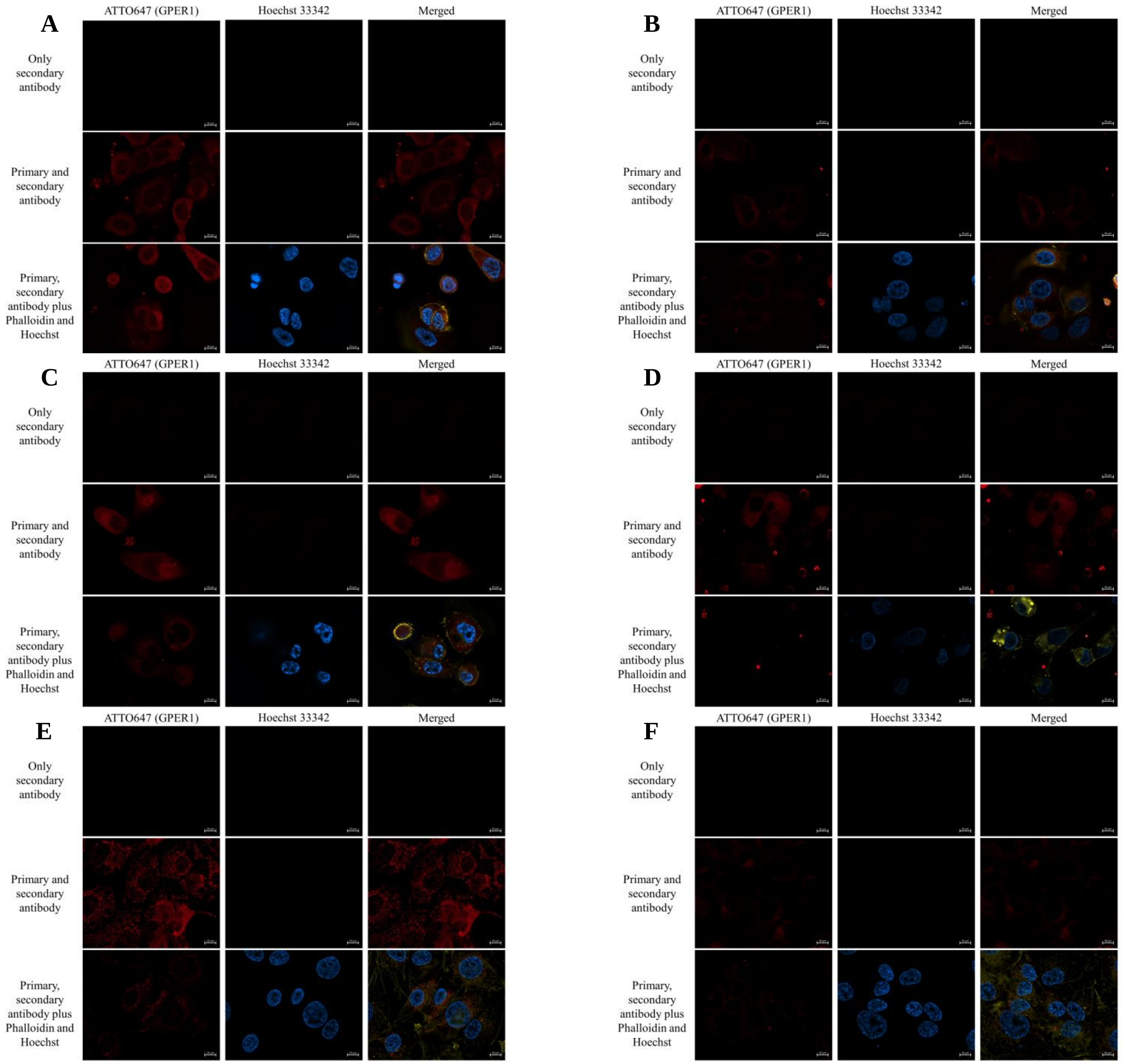
A
ATTO647 (GPER1)
Hoechst 33342
Merged
Only secondary antibody
Primary and secondary antibody
Primary, secondary antibody plus Phalloidin and Hoechst
B
ATTO647 (GPER1)
Hoechst 33342
Merged
Only secondary antibody
Primary and secondary antibody
Primary, secondary antibody plus Phalloidin and Hoechst
C
ATTO647 (GPER1)
Hoechst 33342
Merged
Only secondary antibody
Primary and secondary antibody
Primary, secondary antibody plus Phalloidin and Hoechst
D
ATTO647 (GPER1)
Hoechst 33342
Merged
Only secondary antibody
Primary and secondary antibody
Primary, secondary antibody plus Phalloidin and Hoechst
E
ATTO647 (GPER1)
Hoechst 33342
Merged
Only secondary antibody
Primary and secondary antibody
Primary, secondary antibody plus Phalloidin and Hoechst
F
ATTO647 (GPER1)
Hoechst 33342
Merged
Only secondary antibody
Primary and secondary antibody
Primary, secondary antibody plus Phalloidin and Hoechst

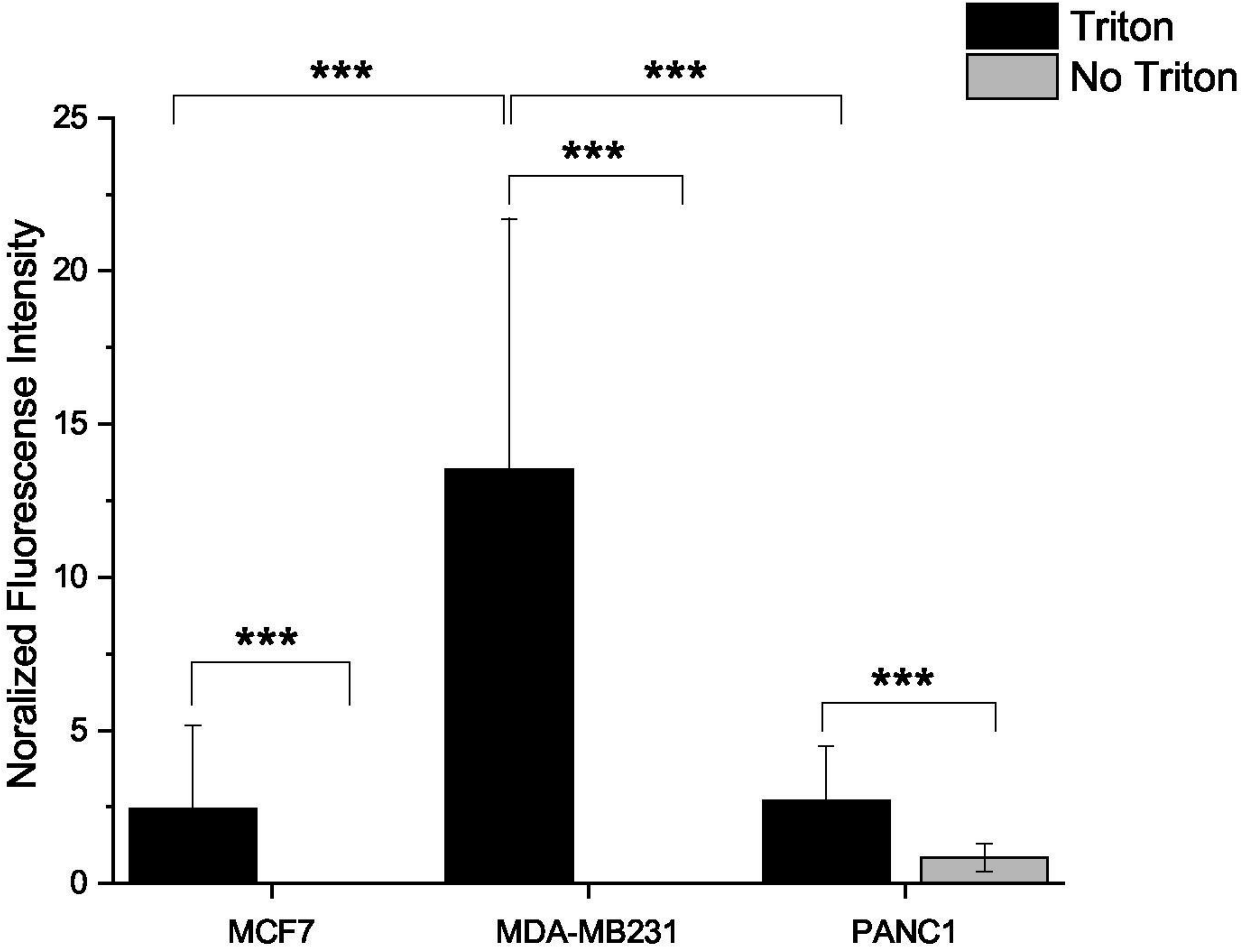
Triton
No Triton
***
***
***
***
***
Noralized Fluorescense Intensity
25
20
15
10
5
0
MCF7
MDA-MB231
PANC1
